# Expanding the Scope of PROTACs: Opportunities and Challenges in Topical Delivery

**DOI:** 10.1021/acs.jmedchem.5c01911

**Published:** 2025-11-19

**Authors:** Laura Gioiello, Rita Maria Concetta Di Martino, Tracey Pirali

**Affiliations:** † Department of Pharmaceutical Sciences, Università degli Studi del Piemonte Orientale, Largo Donegani 2, 28100 Novara, Italy; ‡ Molecular Immunology Unit, Department of Experimental Oncology, Fondazione IRCCS Istituto Nazionale dei Tumori di Milano, 20133 Milan, Italy

## Abstract

Proteolysis-targeting chimeras (PROTACs) have emerged as a novel therapeutic modality primarily through systemic administration. Recently, their topical use has drawn increasing attention, offering a promising strategy to achieve localized protein degradation while minimizing systemic toxicity. This miniperspective highlights key advances in the development of topical PROTACs, exemplified by **AH-001** and **GT20029**, androgen receptor degraders, both in clinical trials for androgenetic alopecia, with **GT20029** also evaluated for acne. We discuss emerging degraders that target clinically relevant proteins, including JAK kinases and BET family members, with applications extending beyond dermatology to pulmonary and ocular diseases and even cosmetic use. Innovative strategies, such as liposomes and microneedle systems, are also enabling effective local delivery. Despite this progress, topical PROTACs face some challenges, such as optimizing the stability, tissue penetration, and selective target engagement under physiological conditions. We conclude by outlining strategic directions that could accelerate the clinical translation of this emerging therapeutic modality.

## Graphical abstract

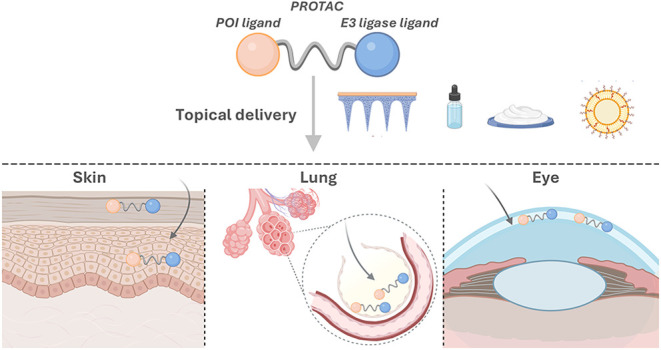

## Introduction

One of the major limitations in pharmacotherapy is the suboptimal efficacy of traditional small molecules, which typically function by transiently occupying the binding site of a target protein. This might lead to incomplete suppression of the protein function and limited effectiveness. Proteolysis-targeting chimeras (PROTACs) offer a transformative solution by catalytically degrading disease-relevant proteins through the ubiquitin-proteasome system (UPS), enabling protein knockdown and a sustained therapeutic effect. This paradigm shift has demonstrated promising efficacy in preclinical and clinical studies, especially in cancer therapy.^1^ Notably, recent Phase III clinical trial results demonstrated that vepdegestrant (also known as ARV-471), a PROTAC targeting the estrogen receptor (ER), prolonged progression-free survival in patients with ER-positive, HER2-negative advanced breast cancer, although the benefit was limited to the subgroup harboring ESR1 mutations and was not observed in the overall patient population.^2^

Despite their therapeutic promise, two major challenges limit the clinical translation of PROTACs. The first limitation is the potential for systemic toxicity. While PROTACs usually provide higher selectivity at the protein level by degrading specific targets,^3^ their systemic distribution and the widespread expression of E3 ligases usually engaged by PROTACs may result in degradation in both diseased and healthy tissues, potentially leading to unwanted toxicity. To overcome this limitation, various strategies, collectively referred to as conditional PROTACs, are emerging to enable spatial, temporal, and spatiotemporal control of target protein degradation; however, many of these approaches are still in their infancy.^4,5^ The second major hurdle for PROTAC development is pharmacokinetics (PK). Due to their large molecular weight (MW), structural complexity, and high polarity, PROTACs often display poor permeability, rapid systemic clearance, and suboptimal oral bioavailability. These properties may necessitate higher doses or parenteral administration, which can increase the risk of adverse effects and complicate long-term patient adherence. Nonetheless, recent studies have identified physicochemical parameters associated with improved oral absorption of PROTACs, which may guide medicinal chemists in the design of orally bioavailable candidates.^6^ Moreover, different delivery systems, including nanocarriers, have been investigated at the preclinical stage to mitigate the PK challenge,^7^ even though the strategies adopted in clinical settings still remain relatively limited.

Topical administration of protein degraders represents a compelling approach to mitigate both issues by restricting the exposure of PROTAC to the site of disease (Figure 1). By delivering the degrader locally, whether to the skin, respiratory tract, or ocular surface, topical application can reduce systemic absorption and toxicity, while bypassing the PK limitations associated with systemic delivery. Moreover, topical application might significantly improve patients’ compliance compared to parenteral administration. Notably, two topically administered PROTACs have accessed clinical trials: **AH-001** ^8^ and **GT20029**,^9^ both androgen receptor (AR)-targeting degraders. **AH-001** is currently in a phase I clinical trial (NCT06927960) for androgenetic alopecia (AGA). **GT20029** has completed phase I studies for AGA and acne (NCT05428449 and NCT06468579) and a phase II trial for AGA (NCT06692465).

**Figure 1:**
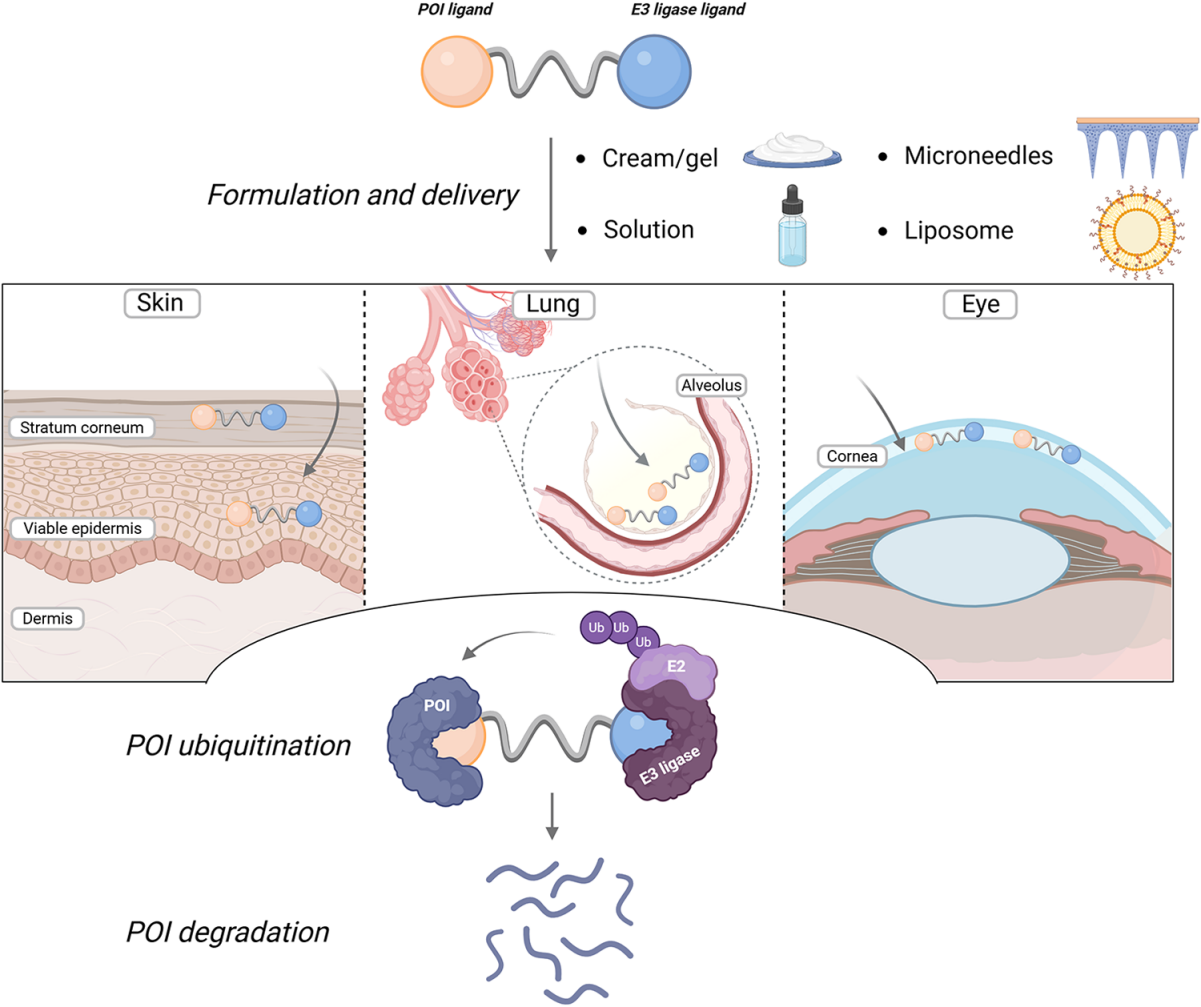
Sites and mechanism of action of topical PROTACs.

In this miniperspective, we will explore recent advances in the development of PROTACs for topical use, highlight emerging therapeutic areas and formulation strategies, discuss the limitations of this approach, and propose future directions to enhance its clinical utility. The purpose of this paper is to encourage further exploration of locally restricted PROTACs and expand their application in clinical therapy. To guide the reader through the discussion, Table 1 summarizes key examples of PROTACs for topical use that are described throughout the manuscript.

**Table 1:** Selected Topical PROTACs and Their Properties^a^

PROTAC	indication	stage	target	*in vitro* potency (DC_50_)	delivery/formulation	preclinical model	comments	reference
**AH-001**	AGA	I	AR		unspecified topical formulation (0.2%, 0.5%, 1%, 2%)		first-in-human study evaluating safety, tolerability, and PK at multiple doses	^8^
**GT20029** ^c^	acne vulgaris and AGA	I	AR		gel		first-in-human study evaluating safety, tolerability, and PK in healthy volunteers	^9,10^
	AGA	II	AR		tincture (0.5%, 1%)		promoted hair growth; no adverse sexual events; planned phase II US and phase III China trials	^9^
Se-AR PROTAC	androgenetic alopecia (AGA)	preclinical	AR	693 nM (C4-2)	Se nanoparticles-loaded hyaluronic acid microneedles	AGA mouse model	peptide-based PROTAC; faster hair regrowth compared to minoxidil; no cytotoxicity in epithelial cells; low systemic exposure	^11^
**TJA-107**	AGA	preclinical	AR	155 nM (HDPC)	hyaluronic acid microneedles	AGA mouse model; AGA recrudescence model	superior hair regrowth in AGA recrudescence model; no cytotoxicity in epithelial cells; no systemic toxicity	^12^
**C6**	AGA	preclinical	AR	262 nM (HDPC)	topical solution (0.1%, 0.4%)	AGA mouse model	optimized derivative of **TJA-107** with improved skin retention; comparable efficacy to 5% minoxidil; high skin-to-plasma concentration ratio indicating low systemic exposure	^13^
**JAPT7**	atopic dermatitis (AD)	preclinical	JAK1/2	JAK1 = 682 nM, JAK2 = 2.32 μM (M1-RAW264.7)	O/W emulsion (0.15%)	AD mouse model	superior reduction of inflammation compared to ruxolitinib (0.75%); no hook effect at high dose	^14^
**JAPT8**	AD	preclinical	JAK1/2	JAK1 = 1.32 μM, JAK2 = 2.36 μM (M1-RAW264.7)	O/W emulsion (0.15%)	AD mouse model	superior reduction of inflammation compared to ruxolitinib (0.75%); no hook effect at high dose	^14^
**PJ-001**	AD	preclinical	JAK2	<100 nM (Jeko-1)	O/W emulsion (2%, 4%)	DNFB-induced AD mouse	antipruritus and anti-inflammatory effects; effective at 2%; hook effect at 4%	^15^
**TD9**	hyperpigmentation	preclinical	TYR	∼50 μM (A375)		zebrafish	levodopa-based degrader; effective melanin reduction; suitable for microneedle delivery	^16^
**D6**	hyperpigmentation	preclinical	unknown			zebrafish	indirect TYR downregulation; more potent antimelanogenic effect than rhein and arbutin *in vivo*	^17^
**D16**	hyperpigmentation	preclinical	pCREB		topical solution	mouse model of induced pigmentation	indirect TYR downregulation via pCREB degradation; reduction of skin darkening *in vivo*; no toxicity in liver and kidneys	^18^
**ERD-308**	breast cancer	preclinical	ERα	0.17 nM (MCF-7)^b^	MPEG-poly(β-amino ester) micelle-loaded methacrylated hyaluronic acid microneedles	murine breast xenograft (MCF-7 derived)	high tumor retention (>87%); combination with palbociclib induced >80% tumor regression; no skin irritation or systemic toxicity observed	^19,20^
**1**	idiopathic pulmonary fibrosis (IPF)	preclinical	BRD4	0.29 nM (NHLF)		C57BL/6 mice	sustained and localized BRD4 degradation in lung tissue; no degradation after IV dosing	^21^
**lipo@** ^**ET**^ **TAG-2**	corneal neovascularization (CNV)	preclinical	LRG1	7 μM (HUVEC)	liposomes	alkali burn- induced CNV mouse model	peptide-based PROTAC; enhanced retention on the corneal surface, and intracellular LRG1 degradation; improved potency and prolonged retention vs free ^ET^TAC-2	^22,23^

a DC_50_ values were determined in the following cell lines: C4-2 (prostate); HDPC (dermal papilla); M1-RAW264.7 (inflammatory macrophage); Jeko-1 (lymphoma); A375 (melanoma); MCF-7 (breast); NHLF (normal human lung fibroblast); HUVEC (human umbilical vein endothelial). For **AH-001** and **GT20029**, the study status for each therapeutic use is indicated according to the latest data from www.clinicaltrials.gov. Study status information as of October 2025.

b DC_50_ was reported for the unencapsulated form.

c **GT20029** met the primary end points in phase II trials for AGA (CTR20230669) and acne (CTR20240799) in China.

## Topical PROTACs for Dermatological Disorders

The skin, while readily accessible, presents a severe barrier to drug delivery. Its outermost layer (i.e., the stratum corneum) forms a dense, lipid-rich matrix that restricts the penetration of most molecules, especially large and polar compounds such as PROTACs. With a typical MW well above 500 Da, and often featuring multiple hydrogen bond donors and acceptors as well as a high TPSA (topological polar surface area), PROTACs lie far outside the traditional “Rule of Five” chemical space. Despite these limitations, several physicochemical parameters have emerged as useful predictors of skin permeation behavior. Moderate lipophilicity (log *P* ∼1–3), low melting point (<200 °C), which is related to an appropriate solubility, and balanced hydrogen bonding capacity can facilitate both stratum corneum partitioning and diffusion through the epidermis.^24,25^ However, no single property guarantees skin permeation; rather, an integrated physicochemical profile is required. For example, rigid or bulky molecular structures in PROTACs may hinder intercellular diffusion, while high polarity can limit partitioning into the lipid matrix. Conversely, highly lipophilic degraders may exhibit strong retention in the outer skin layers but poor permeation into deeper layers, where many targets reside.

To overcome these challenges, linker and E3 ligase ligand engineering has proven critical: small changes in flexibility, ring strain, and polarity can significantly impact not only dermal penetration and degradation efficiency but also stability, as seen in recent studies on AR degraders.^13^ Furthermore, advances in delivery technology, such as microneedles and nanoparticles, offer practical solutions to bypass or modulate the stratum corneum barrier. These platforms can be tailored to achieve both a high local concentration and sustained release within the epidermis or hair follicles.

The following section highlights the advances in this area, showcasing how the rational design of both degraders and formulation can overcome the intrinsic limitations of topical PROTAC delivery.

### PROTACs for Androgenic Alopecia

Androgenetic alopecia (AGA) is a chronic condition characterized by hair loss on the scalp, affecting both men and women, although with a higher prevalence in males. Its pathogenesis is multifactorial, with substantial evidence linking abnormal AR activity to the onset and progression of the disease.^26^ Although oral AR antagonists have been proven to be effective in promoting hair regrowth, systemic exposure to these agents is associated with significant adverse effects, including male sexual dysfunction. As a result, topical AR antagonists are considered a more appropriate therapeutic approach for AGA, but no such compounds have yet received clinical approval for this indication.^27^ Clascoterone, a topical soft drug approved in 2020 for acne,^28^ is a competitive AR antagonist currently being evaluated in phase III (NCT05910450 and NCT05914805) trials for AGA. In this context, topical AR-targeting PROTACs have been attracting increasing interest, especially when combined with microneedle patch technology.

Microneedle patches represent an innovative drug delivery system that uses microscale needles to penetrate the stratum corneum barrier, enabling the targeted delivery of substances to the epidermis or upper dermis. This technology offers remarkable versatility, as it can be used to convey drugs of various types, including small molecules, inorganic compounds, cytokines, and biopharmaceuticals. Moreover, microneedles can be thoroughly customized in terms of shape, size, material, and mode of administration, offering a highly tailored solution to specific needs and diseases.^29^ This delivery is particularly advantageous for PROTACs, which may exhibit limited passive skin penetration. In addition, when treating AGA, microneedles form a drug reservoir in the layers where most hair follicles reside, allowing for site-specific drug delivery while overcoming the penetration limitations typically associated with PROTACs.

By employing artificial intelligence-assisted drug design, the group led by Lei Li developed **Se-AR PROTAC**, a peptide-based degrader capable of degrading AR by forming a ternary complex with the von Hippel–Lindau (VHL) E3 ligase, with a DC_50_ of 693 nM in AR-positive prostate cancer cells (Figure 2). The PROTAC is composed of three main components: an AR recognition motif, a linker peptide, and a VHL-recognition motif that enables E3 ligase recruitment. In addition, a short peptide sequence was added to facilitate coupling with selenium nanoparticles. Selenium was specifically chosen for its ability to scavenge reactive oxygen species (ROS), which are known to be key contributors to hair loss.^30^ Due to the high hydrophilic nature of the peptide and the consequent low affinity with the stratum corneum, **Se-AR PROTAC** was administered to the AGA mouse model via dissolvable microneedles composed of hyaluronic acid (HA) for the skin-soluble portion and polyvinylpyrrolidone K90 for the detachable backing layer. This multifunctional formulation induced the simultaneous (i) degradation of AR, (ii) elimination of ROS, (iii) enhancement of local microvasculature, and (iv) mechanical skin stimulation. Compared to minoxidil, an AGA approved topical vasodilator that stimulates follicles by increasing blood flow, **Se-AR PROTAC** allowed for earlier skin pigmentation (an indicator of hair follicle activation), increased angiogenesis, and regeneration of hair follicle cells, ultimately resulting in successful hair regrowth. It showed no toxicity in normal epithelial cells and, notably, when tested *in vivo*, remained predominantly localized within the skin, with minimal systemic exposure and no signs of hematological, renal, or hepatic toxicity.^11^

**Figure 2:**
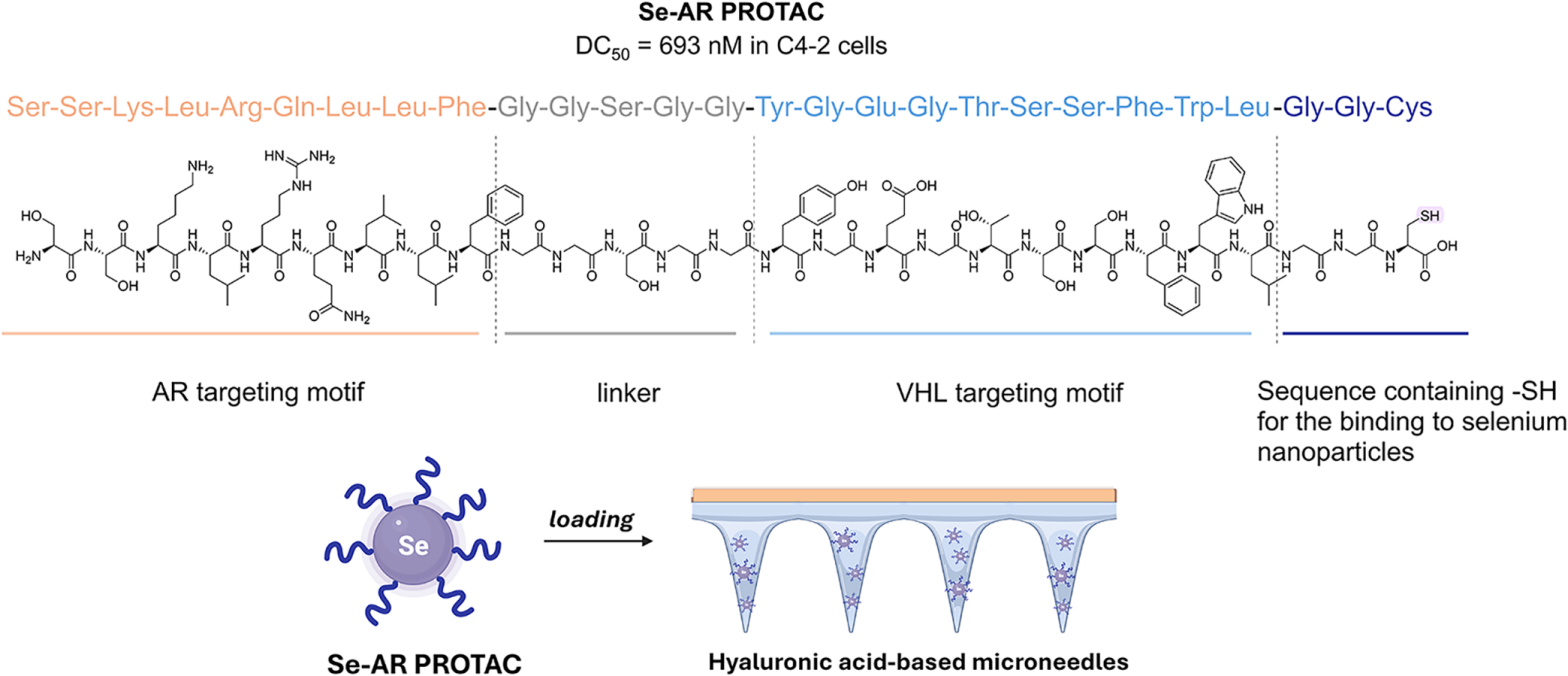
Design and delivery of **Se-AR PROTAC**.

In addition to peptide-based PROTACs, the same microneedle delivery technology has also been applied to enhance the skin retention of **TJA-107**, a small molecule-based AR PROTAC degrader (Figure 3). **TJA-107** was assembled by linking an AR antagonist to an E3 ligase Cereblon (CRBN) ligand. The POI ligand is a derivative of the AR antagonist enzalutamide and similar to that of bavdegalutamide (ARV-110) developed for prostate cancer.^31^ While **TJA-107** demonstrated remarkable potency (DC_50_ = 155 nM) *in vitro*, it was totally inactive when topically administered via tinctures due to its poor skin retention (retention rate = 0.69 ± 0.16%). To overcome this, **TJA-107** was encapsulated within dissolvable HA-based microneedles. After a single administration, this formulation enabled effective transport of the PROTAC across the stratum corneum, leading to hair regrowth in an AGA mouse model and in an AGA recrudescence mouse model, with a faster onset of effect and better hair regrowth after redepilation compared to minoxidil. The PROTAC demonstrated no cytotoxicity in epithelial cells (e.g., HDPCs, human splenic fibroblasts (HSF), human keratinized epidermal cells (HaCaT)) and high biocompatibility *in vivo*, with no histological alterations or variations in AR expression pattern observed in male sexual organs.^12^

**Figure 3:**
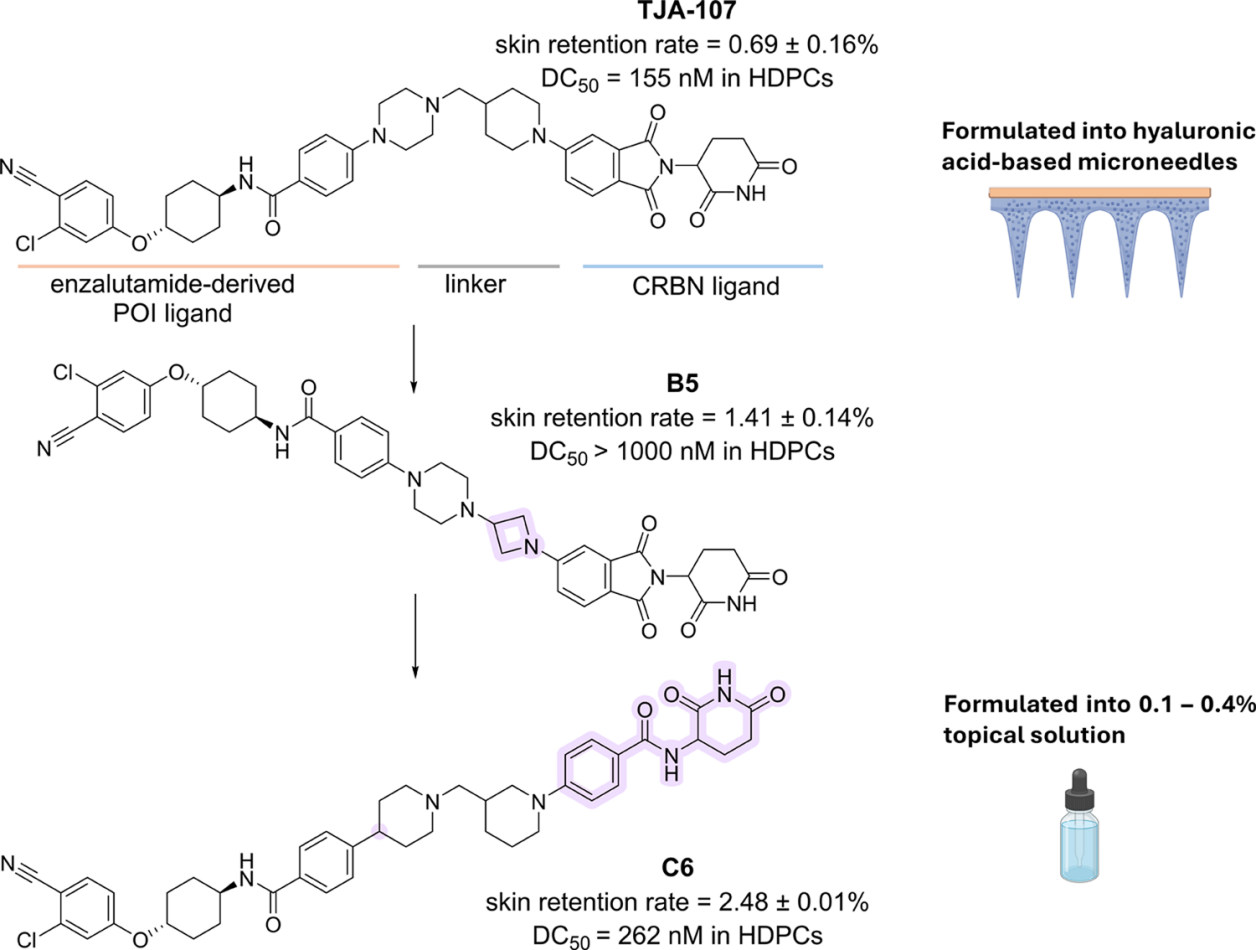
Discovery and optimization of **C6**, an improved analogue of **TJA-107**, with enhanced skin retention.

Although it is less invasive than other delivery methods, microneedle technology still disrupts skin integrity and may still cause temporary inflammation, allergic reactions, or even infections.^32^ In addition, microneedles have a limited surface area or matrix volume, which constrains the amount of drug that can be delivered, a significant limitation for large molecules requiring high local concentrations such as PROTACs. Alternatively, careful optimization of the PROTAC physicochemical properties may allow for effective topical administration without relying on such invasive delivery systems. **TJA-107** was modified by introducing tailored modifications to increase the skin retention rate. By testing a series of model compounds with various linkers and E3 ligase ligands, the authors showed that the substitution of piperidine with azetidine and, therefore, the increase in ring strain and overall linker rigidity augmented skin retention, as exemplified by compound **B5** (Figure 3). However, this modification was not maintained as it negatively affected degradation activity. The optimized candidate, **C6**, exhibited a DC_50_ of 262 nM in HDPC cells and a skin retention rate of 2.48 ± 0.01%, almost 4-fold higher than its precursor **TJA-107** (Figure 3). This result was achieved by (i) decreasing TPSA by removing nitrogen atoms from the linker and (ii) replacing lenalidomide or thalidomide with a benzamide analogue, which also has a lower TPSA and higher clogP. In AGA mouse model, topical administration of **C6** solution at 0.4% was as effective as 5% minoxidil solution in promoting hair regrowth and, remarkably, its skin accumulation was significantly higher than plasma and other tissues (ratio of skin to plasma drug concentration at 12 h = 1736.28; ratio of skin to lung drug concentration at 12 h = 288.26), suggesting a promising safety profile with reduced systemic exposure.^13^

The potential of a targeted AR degradation approach for the topical treatment of AGA is corroborated by the use of PROTACs currently in clinical trials. **AH-001** (structure undisclosed), developed by AnHorn Medicines, is being evaluated in a phase I study (NCT06927960), aiming at evaluating the safety, tolerability, and PK of single and multiple ascending doses at concentrations of 0.2%, 0.5%, 1%, and 2% in healthy volunteers and male subjects with AGA.^8^ Another AR-targeting PROTAC in a more advanced stage is **GT20029** (structure undisclosed), developed by Kintor Pharma and currently under clinical evaluation for two dermatological indications: AGA and acne vulgaris. For AGA, administered as a 0.5% or 1% tincture, the compound successfully met the primary end points in a phase II Chinese trial (CTR20230669), with the U.S. counterpart registered as NCT06692465.^9^ Specifically, **GT20029** promoted hair growth with significant therapeutic efficacy and clinical significance compared to the placebo, without causing adverse sexual events. Based on these results, the compound is expected to advance to a phase II clinical trial in the United States and a phase III trial in China. For acne vulgaris, **GT20029** has also been used in clinical testing. Two phase I trials (NCT05428449 and NCT06468579) evaluated its safety, tolerability, and PK in healthy volunteers, while a phase II gel trial in China (CTR20240799) has reportedly reached its primary end point (with 0.5% dose recommended for phase III).^10^

### PROTACs for Atopic Dermatitis

The Janus kinase/signal transducer and activator of transcription (JAK/STAT) pathway is one of the main signaling cascades activated by cytokine stimulation and is crucial for many biological processes, including cell proliferation, inflammation, apoptosis, and immune regulation. In detail, JAKs are a family of tyrosine kinases composed of four members, JAK1, JAK2, JAK3, and tyrosine kinase 2 (TYK2) that, upon activation, phosphorylate the family of STAT transcription factors. This signaling axis is activated by over 50 different cytokines, each binding to its specific receptor, thereby triggering the activation of multiple JAK isoforms. Notably, a single JAK can be recruited by different cytokine receptors and, in turn, activate various STAT subtypes, ultimately leading to a staggering variety of cellular responses. It comes as no surprise that a dysregulation of this mechanism is associated with numerous disorders, including allergic and autoimmune conditions.^33^ Among these, atopic dermatitis (AD) is a chronic inflammatory skin disease characterized by the disruption of epidermal barrier function and dysregulation of Th2 immune response, causing symptoms such as severe dryness, intense itching, and epidermal desquamation.^34^ By blocking the signaling cascade triggered by cytokine overstimulation, JAK inhibitors like abrocitinib, upadacitinib, and baricitinib help reduce inflammation and restore the skin barrier. However, due to the overlapping roles of JAK isoforms in multiple cytokine pathways and the lack of isoform selectivity, systemic administration of JAK inhibitors often results in significant adverse effects. These include reactivation of latent infections, hematopoietic alterations, malignancies, as well as increased cardiovascular and thromboembolic risks,^35^ which prompted the US Food and Drug Administration (FDA) to issue a black box warning.^36^

Hence, there is a high demand for new drugs with enhanced organ selectivity to minimize unnecessary systemic exposure and reduce the risk of serious side effects. In this context, ruxolitinib, the first topical JAK inhibitor approved for AD, serves as a clinically validated example of the benefits of the topical approach.^35^ Furthermore, topical soft drugs that are active in the skin with minimal systemic exposure due to the presence of soft spots targeting JAKs have been developed,^37^ such as CEE321.^38^ In the pursuit of novel topical agents for AD, the group led by Zhongjian Chen explored the therapeutic potential of **JAPT7** and **JAPT8**, two PROTACs targeting JAK1 and JAK2 previously developed for the treatment of lymphoblastic leukemia. These compounds are composed of CRBN ligands as E3 ligase ligands and baricitinib (**JAPT8**) or its derivative (**JAPT7**) as the POI ligands. In both cases, the POI ligands were modified by incorporating a 4-amino-benzamide moiety onto the solvent-oriented C2-carbon of the pyrimidine core as a linker attachment point (Figure 4).^39^ **JAPT7** and **JAPT8** were investigated for their anti-inflammatory effects in RAW264.7 cells, a murine macrophage cell line that the authors polarized into the pro-inflammatory M1 phenotype. Here, thanks to their ability to degrade JAK1 and JAK2 (**JAPT7** with DC_50_ of 682 nM (JAK1) and 2.32 μM (JAK2), and **JAPT8** of 1.32 μM (JAK1) and 2.36 μM (JAK2)), both compounds demonstrated to downregulate AD-related inflammatory cytokines, such as interleukin (IL)-4, IL-13, IL-33, and thymic stromal lymphopoietin. While most PROTACs exhibit a hook effect at high concentrations, **JAPT7** and **JAPT8** maintained their activity, even at higher doses. Notably, in a murine model of AD, topical formulations of **JAPT7** and **JAPT8** (each at 0.15%) outperformed ruxolitinib (0.75%) in ameliorating AD symptoms. Specifically, compared to the JAK inhibitor, the PROTACs more effectively (i) reduced erythema and improved skin smoothness; (ii) restored keratinization; (iii) reduced mast cell infiltration; (iv) downregulated pro-inflammatory mediators such as ILs, interferon (IFN)-γ, and immunoglobulin E (IgE); and (v) reduced immune cell expansion and systemic inflammation, as reflected by the normalization of spleen size and morphology.^14^

**Figure 4:**
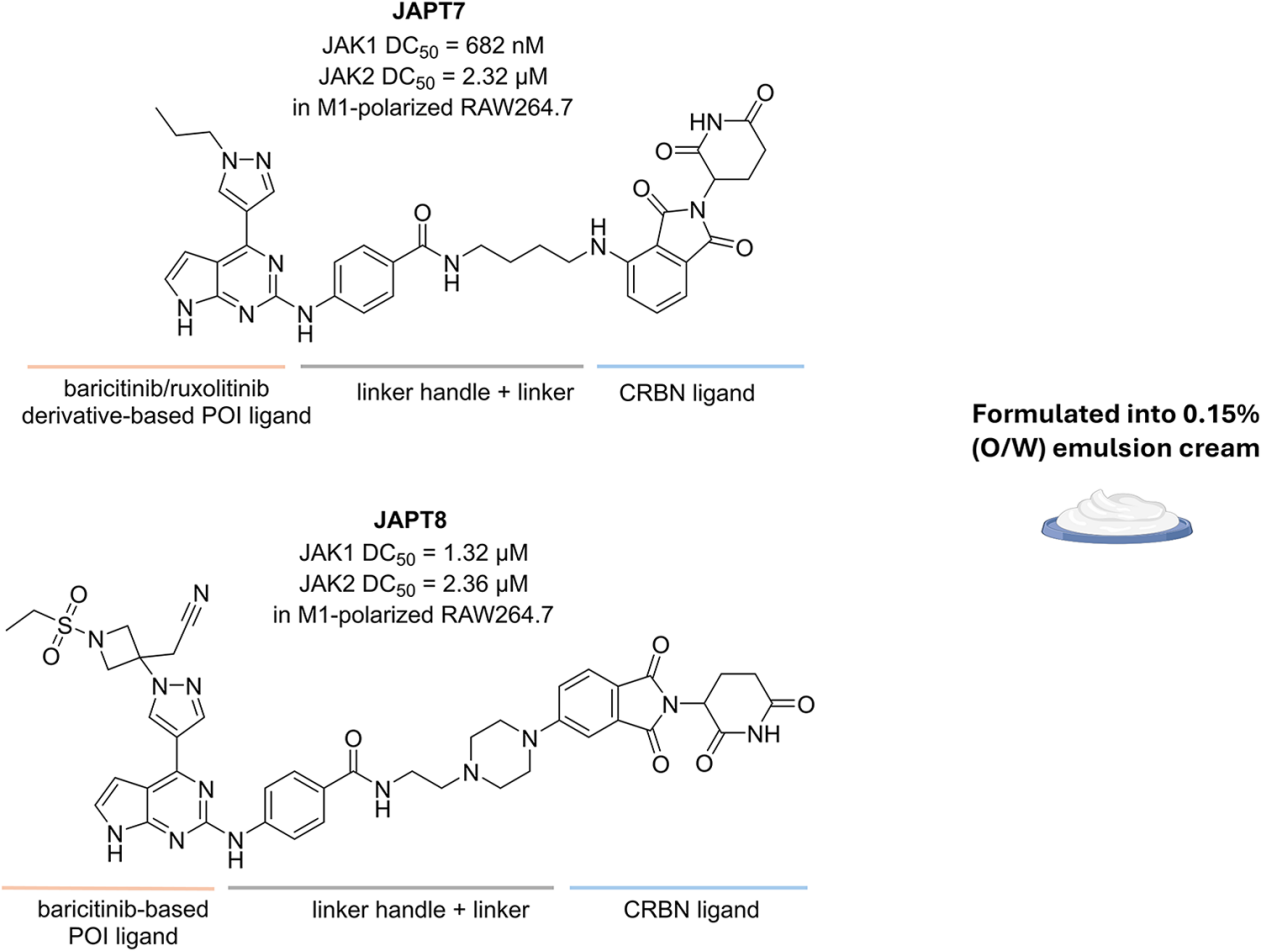
Structures and formulation of **JAPT7** and **JAPT8**.

Another promising topical PROTAC for AD is **PJ-001** (structure undisclosed), which catalyzes the degradation of JAK2 in Jeko-1 cells with a DC_50_ < 100 nM exploiting the CRBN E3 ligase. The efficacy of **PJ-001**, formulated as 2% and 4% O/W emulsions, was evaluated in a 2,4-dinitrofluorobenzene (DNFB)-induced mouse model of AD. As evidenced by the reduction of the number of scratches on mice skin, **PJ-001** showed antipruritus properties. Moreover, it was as effective as dexamethasone, a corticosteroid, and crisaborole, a soft PDE4 inhibitor approved for mild-to-moderate AD,^40^ in reducing histopathological features of the disease, like hyperkeratosis and inflammatory cell infiltration. These effects were particularly pronounced with the 2% formulation, likely due to a hook effect observed at higher concentrations. Furthermore, **PJ-001** treatment modulated the expression of markers associated with inflammation and autophagy (such as NF-κB, LC3, and Beclin 1) and significantly upregulated filaggrin, indicating enhanced skin barrier function.^15^

It is important to note that JAK2 mediates the signaling of cytokines such as granulocyte colony-stimulating factor and erythropoietin, which regulate myelopoiesis and erythropoiesis; therefore, its systemic inhibition is associated with adverse effects such as neutropenia, anemia, and thrombocytopenia.^35^ Hence, the topical delivery of PROTACs represents a promising strategy to modulate JAK2 locally in the skin, with the potential to avoid systemic hematological side effects.

### PROTACs for Pigmentary Skin Disorders

Melanin plays an important role in protecting the skin from UV radiation, pollutants, and toxic chemicals and is produced by melanocytes during the pigmentation process. However, melanin overproduction is related to dermatological conditions like melasma, ephelides, sunspots, pigmented acne scars, and even skin cancer.^41^ The rate-limiting step of the biosynthesis of melanin is represented by the hydroxylation and oxidation of l-tyrosine to dopaquinone, catalyzed by the enzyme tyrosinase (TYR). Current depigmenting agents like hydroquinone, kojic acid, arbutin, l-ascorbic acid, and ellagic acid work by inhibiting TYR. However, apart from requiring several months to years of treatment to be effective, they have significant drawbacks: for example, hydroquinone displays notable toxicity, while arbutin and l-ascorbic acid are chemically unstable.^42^ These limitations have prompted research into safer and more effective skin-whitening modalities.

**TD9** is the first TYR-targeting PROTAC reported in the literature and is based on levodopa as the POI ligand and a CRBN binder as the E3 ligase ligand (Figure 5). Capitalizing on the resolved cocrystal structure of TYR with levodopa, the carboxyl group of levodopa was exploited as the linker attachment point, since it extends toward the solvent-exposed region of the protein. From a SAR study around the linker, **TD9** emerged as the most active degrader with a DC_50_ of approximately 50 μM in A375 cells, and it also inhibits TYR with an IC_50_ of 113 μM.^16^ Nevertheless, it remains conceivable that **TD9** may induce the degradation of additional off-target proteins, particularly the α- and β-adrenergic receptors to which levodopa is known to bind. Therefore, **TD9** was tested in A375 cells, and it did not alter α- or β-adrenergic receptor levels, likely due to steric hindrance between these receptors and the E3 ligase when forming the ternary complex. These findings further confirm a key advantage of PROTAC technology over traditional inhibitors: even a promiscuous ligand can be converted into a highly selective degrader.^3^ To further validate its activity *in vivo*, the PROTAC was tested in a zebrafish model, whose tyrosinase is highly homologous to the human enzyme. After 48 h of treatment with 25–100 μM **TD9**, melanin levels were significantly decreased, and notably, this depigmenting effect was maintained even after 96 h, the time at which the pigmentation had returned to normal in zebrafish treated with kojic acid. To envisage the feasibility of topical application, the authors investigated the physicochemical properties of **TD9**: (i) its MW is less than 540 Da; (ii) it is more soluble in water compared to kojic acid and ellagic acid (11.4 mg/mL vs 0.055 and 0.00097 mg/mL, respectively); and (iii) it is highly hydrophilic, with a log *P* of −0.65. Its high hydrophilicity indicates that a microneedle patch formulation could be the most suitable strategy to enhance penetration through the stratum corneum and improve skin retention, although this delivery approach was not explicitly explored by the authors.^28^

**Figure 5:**
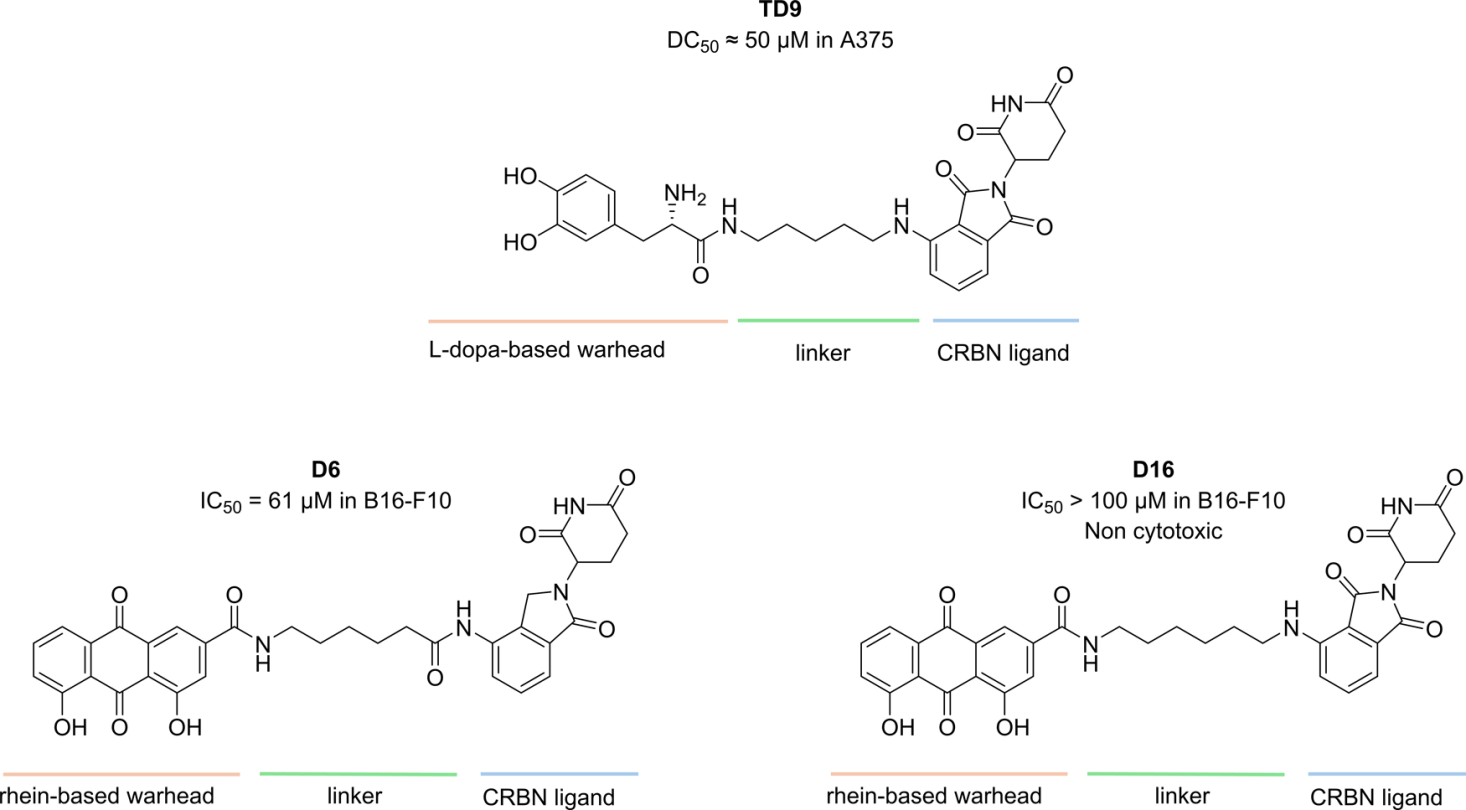
Structures of **TD9**, **D6**, and **D16**.

Although TYR is constitutively expressed in melanocytes, its expression can be further induced by stimuli, such as UV radiation or hormones. Specifically, these stimuli activate keratinocytes, which, through a signaling cascade involving mediators such as phosphorylated cyclic adenosine monophosphate-response element binding protein (pCREB), lead to the activation of microphthalmia-associated transcription factor (MITF). The latter then translocates into the nucleus and induces the expression of TYR.^43^ Accordingly, PROTACs designed against TYR upstream modulators hold potential as effective whitening agents. To this aim, Jieqing Liu’s team developed **D6**, a PROTAC degrader bearing rhein as the POI ligand, an anthraquinone compound naturally found in herbs such as Cassia seeds and *Aloe vera* and commonly used in traditional Chinese medicine (Figure 5). **D6** reduced MITF levels at 25 μM (DC_50_ not reported), which in turn led to a significant reduction in TYR expression. Still, it remained unclear whether the observed reduction in MITF levels resulted from its direct degradation or from the modulation of upstream regulators. In addition, despite showing a stronger antimelanogenic effect in zebrafish compared to rhein as such and arbutin at the same concentration, its development was hampered by a notable cytotoxicity in both keratinocytes (HaCat) and mouse melanoma cells (B16–F10).^17^

Therefore, to overcome this limitation, the authors synthesized **D16**, a derivative of **D6** with an improved tolerability profile. Specifically, the introduction of an alkane linker and the use of pomalidomide as the E3 ligase ligand markedly reduced cytotoxicity (**D6**: IC_50_ = 61 μM; **D16**: IC_50_ > 100 μM in B16-F10 cells), while also enhancing TYR downregulation (Figure 5). Subsequent mechanistic studies, employing techniques such as Cellular Thermal Shift Assay (CETSA), clarified the mode of action: **D16** does not directly degrade TYR, but rather induces proteasomal degradation of its upstream regulator pCREB (DC_50_ not reported), thereby reducing MITF and TYR expression. Of note, when applied topically before UVB irradiation in a mouse model of induced pigmentation, **D16** significantly reduced skin darkening, thus exerting a sunscreen-like protective effect without detectable toxicity in the liver and kidneys.^18^

### PROTACs for Cancer Therapy

Although still in preclinical or early clinical stages, microneedle patch technology has also been applied to the treatment of various types of cancer, particularly those that are superficially accessible, such as skin cancer, oral squamous cell carcinoma, and breast cancer. This approach not only enhances the accumulation of chemotherapeutic and immunotherapeutic agents directly within tumors but also helps reduce the common systemic side effects associated with these treatments.^44^

**ERD-308** is a PROTAC featuring a raloxifene-derived POI ligand and VHL ligand as the E3 ligase ligand, designed to degrade ERα, with a DC_50_ of 0.17 nM in MCF-7 cells (Figure 6).^20^ Thanks to microneedle technology, it proved promising therapeutic potential for ER-positive breast cancer after topical administration. In particular, to improve its targeted delivery and antitumor efficacy, it was coencapsulated with the FDA-approved cyclin-dependent kinase (CDK)­4/6 inhibitor palbociclib into pH-sensitive micelles composed of MPEG-poly­(β-amino ester) block copolymers, specifically designed to disassemble and release their drug payload in response to the mildly acidic tumor microenvironment. After validating efficient cellular uptake in MCF-7 cells and confirming the absence of cytotoxicity in nontumoral HUVECs and HEK293T cells, the micelles were loaded into cross-linked methacrylated HA-microneedle patches and applied directly onto the skin at the breast tumor site in a murine xenograft model. Thanks to this formulation, over 87% of the drug content was retained in the tumor, and a single application was enough to extend ERα degradation from 1 day, as seen with oral dosing, to at least 4 days. Notably, after 4 weeks of treatment on a 4 day-basis, micelles containing only palbociclib inhibited tumor growth, whereas microneedle patches codelivering **ERD-308** and palbociclib induced over 80% tumor regression. This outcome is likely the result of a synergistic mechanism involving ER degradation and CDK4/6 inhibition, which converges on the suppression of the key cell-cycle regulator E2F1 and the induction of apoptosis. Importantly, the treatment was well tolerated: no skin irritation or inflammation was observed at the administration site and, in contrast to oral administration, no significant alterations in liver function or blood glucose levels were reported in the preclinical model.^19^

**Figure 6:**
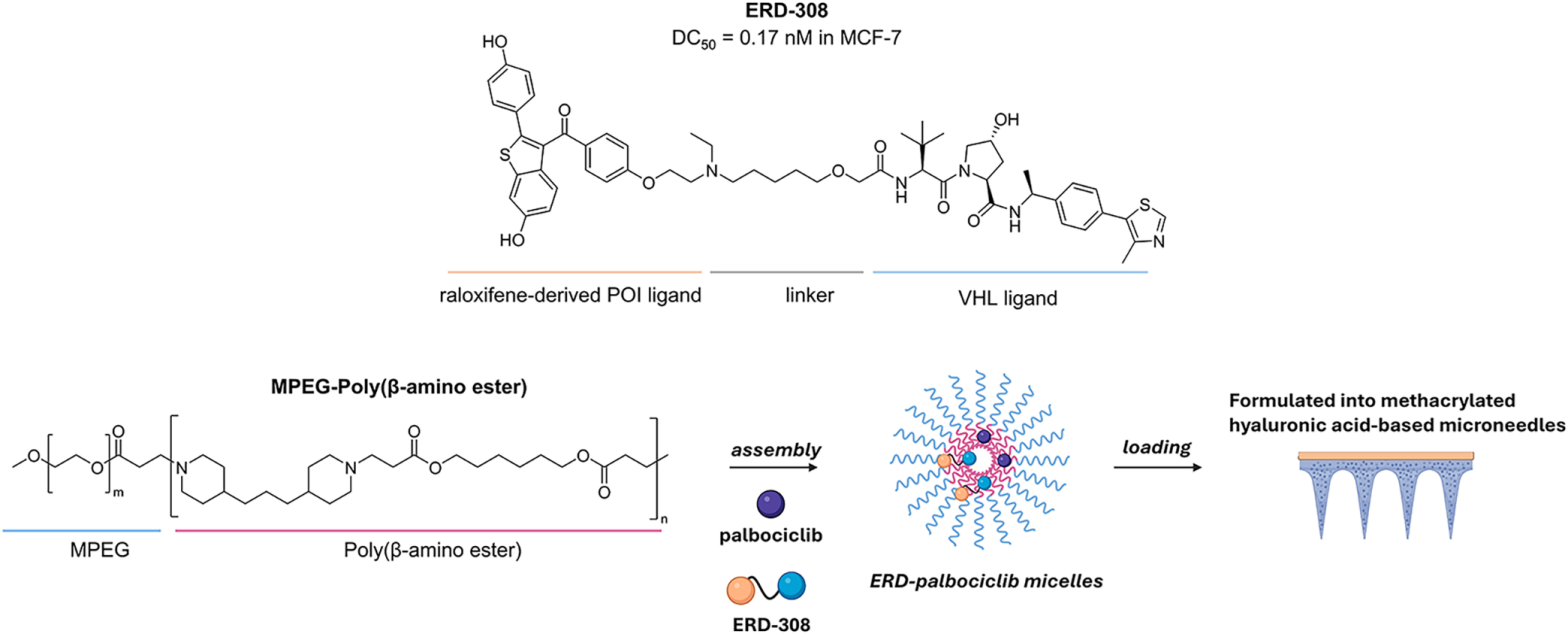
Structure of **ERD-308** and incorporation into MPEG-poly­(β-amino ester) micelles-loaded microneedles.

## Inhaled PROTACs for Pulmonary Diseases

Beyond dermatological conditions, the principles of localized protein degradation are now being extended to the respiratory tract, where inhaled PROTACs may offer a novel approach to treating fibrotic and inflammatory lung diseases. Inhalation enables the direct delivery of degraders to the site of pathology, improving the therapeutic index by maximizing local concentration while minimizing systemic exposure. These features are particularly attractive in chronic pulmonary conditions such as idiopathic pulmonary fibrosis (IPF), where systemic therapies are often limited by toxicity. However, pulmonary delivery poses its own set of challenges: only a fraction of the administered dose typically reaches the lungs (i.e., the respirable fraction), and local drug retention depends on a delicate interplay between particle dissolution, cellular uptake, mucociliary clearance, and macrophage-mediated elimination.^45,46^ Interestingly, several intrinsic features of PROTACs, such as high MW and low solubility, may support lung retention by promoting slow dissolution in lung fluid. Moreover, moderate basicity due to the presence of basic groups, for instance in the linker, may favor accumulation in acidic organelles like lysosomes. Finally, the “hit-and-run” pharmacology of PROTACs, where transient exposure can lead to sustained protein knockdown,^47^ may align well with the pharmacokinetic profile achievable through inhaled delivery.

Encouraged by these considerations and by the success of PROTACs addressing the skin,^14^ the first examples of CRBN-recruiting PROTACs targeting bromodomain and extra-terminal domain (BET) were developed for inhalation therapy in peripheral fibrotic IPF. This condition is a chronic lung disease characterized by progressive scarring of the lungs, which often has a peripheral distribution. Modulation of bromodomain-containing protein 4 (BRD4), either through siRNA-mediated silencing or pan-BET inhibitors, has been shown to halt fibrotic processes in lung-derived cells, including fibroblasts and epithelial cells, as well as in multiple animal models of lung remodeling.^48^ Nevertheless, clinical trials with oral BET inhibitors have raised safety concerns, underscoring the need for safer and more selective BET-targeting strategies. To overcome these limitations, inhaled BET-targeting PROTACs were developed by linking a CRBN ligand, such as lenalidomide or a chemically stable indolyl-dihydrouracil (DHU), to a benzimidazole-based BET inhibitor, selected as the POI ligand following a comprehensive literature review (Figure 7).^49^ Two main strategies were pursued to favor retention in lung tissue: (i) PROTACs (e.g., compounds **1** and **2**) characterized by low aqueous solubility (Sol pH 7.4 < 0.16 μM), allowing administration as a suspension, and (ii) basic PROTACs (e.g., compounds **3** and **4**) containing a piperazine moiety and exhibiting higher solubility (Sol pH 7.4 = 12 μM for **3** and 2.6 μM for **4**), enabling solution-based formulation, with lung retention driven by basicity-induced accumulation in lysosomes.^21^ Moreover, in PROTAC **4**, an ester function was incorporated as a soft spot to promote hydrolysis and further limit systemic exposure, a strategy that has already shown preclinical success in the development of treatments for respiratory diseases.^50^ Among them, PROTAC **3** was not advanced into inhaled PK evaluation due to its low propensity to rapid systemic clearance, while PROTAC **4**, administered intratracheally in mice as a solution, was discarded as it was not detected in lung tissue.

**Figure 7:**
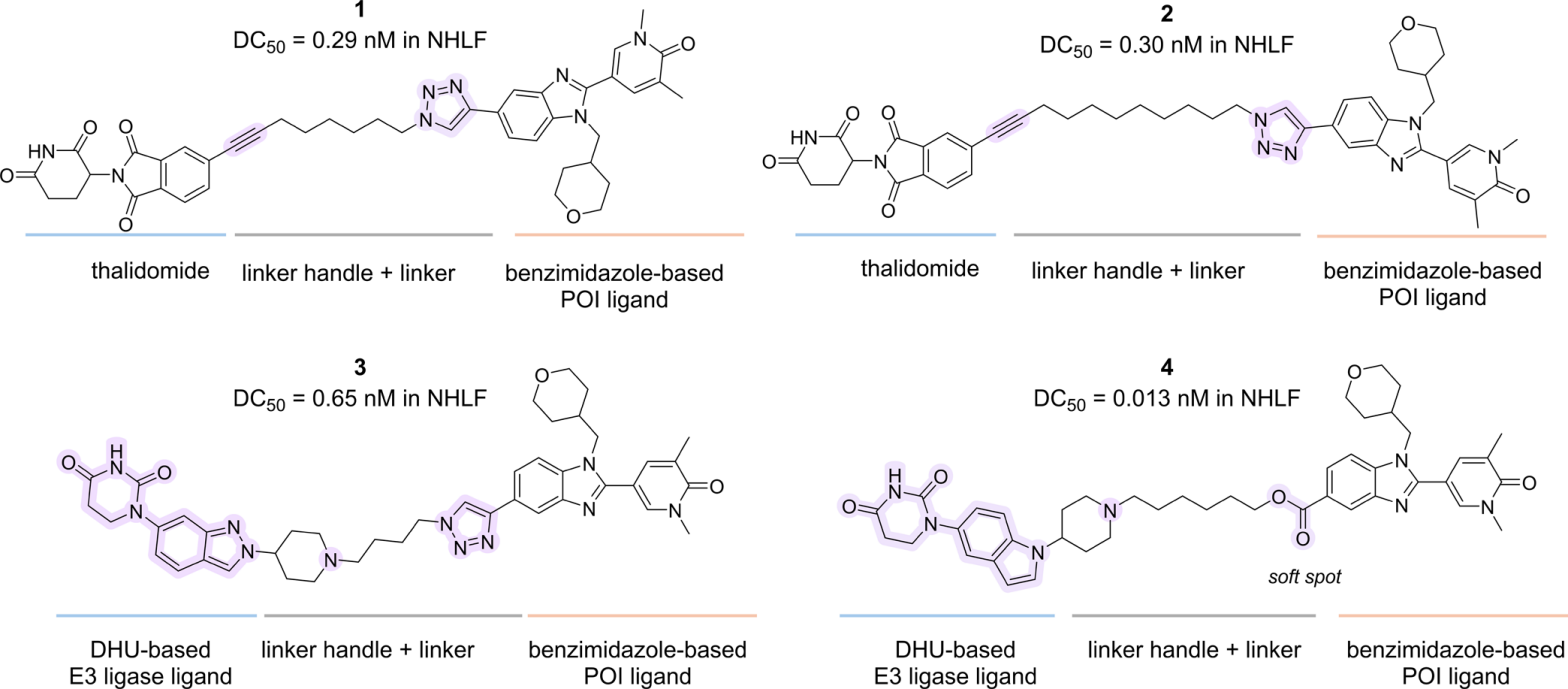
Structure of BET-targeting PROTACs **1–4**.

In an *in vivo* study in mice, although the DC_50_ values in NHLF cells were comparable, **1** outperformed **2** in inducing a substantial and sustained reduction of BRD4 following intratracheal delivery, with effects lasting up to 24 h postdosing. Notably, BRD4 degradation was not observed after intravenous administration of **1**, suggesting a localized lung-specific effect. This was further supported by a dose–response study showing consistent target engagement across lung regions, including the alveolar space, with no detectable spillover into the spleen. These findings highlight **1** and similar strategies as a new therapeutic opportunity for distal airway diseases, where systemic therapies are often limited by narrow safety margins.^21^

## PROTACs for Ocular Application

The human eye is a sensitive and complex organ essential for vision, yet its intricate architecture poses difficult challenges to effective drug delivery. Multiple physiological barriers, including the tear film, cornea, conjunctiva, and blood-ocular barrier, limit drug penetration into targeted ocular tissues. Although numerous potent drugs are available for the treatment of ocular disorders, such as diabetic retinopathy, age-related macular degeneration, glaucoma, and dry eye syndrome, their therapeutic efficacy is often hampered. Topical administration remains the most common route, accounting for over 95% of marketed ophthalmic formulations due to its noninvasive nature. However, this approach suffers from very low bioavailability (less than 5%), primarily as a result of limited corneal permeation, rapid tear turnover, blinking, nasolacrimal drainage, and systemic absorption. As a consequence, frequent administration of high drug doses is typically required, increasing the risk of adverse effects and reducing patient compliance. In response to these limitations, significant efforts have been devoted to the development of nanotechnology-based drug delivery systems that can overcome ocular barriers, protect the drug from degradation, prolong residence time, and enhance corneal penetration.^51,52^

Beyond the development of advanced drug carriers, PROTAC technology has emerged to mitigate corneal neovascularization (CNV) through the selective degradation of leucine-rich α-2-glycoprotein 1 (LRG1). CNV is marked by the abnormal growth and migration of vascular endothelial cells, resulting in the formation of immature blood vessels that sprout from the limbus (i.e., the junction between the cornea and sclera) and invade the corneal stroma, a normally transparent and avascular tissue. This pathological vascularization compromises corneal clarity, disrupts the delicate ocular architecture, and ultimately leads to visual impairment. LRG1 is a secreted glycoprotein of the leucine-rich repeat family implicated in the onset and progression of a wide range of pathological conditions. It plays a central role in angiogenesis, inflammation, fibrosis, and tumorigenesis. In the context of CNV, LRG1 expression is markedly elevated and has emerged as a promising therapeutic target for ocular neovascular diseases, owing to its critical function in promoting pathological vessel growth through activation of the transforming growth factor-β (TGF-β) accessory-Smad1/5/9 signaling cascade. While blockade of LRG1 has shown potential in disrupting this pathway, current therapeutic approaches remain largely limited to antibody-based modalities, highlighting the urgent need for novel and more versatile strategies to effectively target LRG.^22^ In the face of this challenge, a novel PROTAC, ^**ET**^ **TAC-2**, was designed to specifically degrade LRG1 (Figure 8). Originally developed for the treatment of renal fibrosis, its therapeutic scope was expanded to target LRG1 in a murine model of alkali burn-induced CNV, a widely accepted experimental model for studying pathological ocular angiogenesis. ^**ET**^ **TAC-2** is composed of an LRG1-targeting ET peptide linked via a flexible spacer to a lenalidomide-based CRBN-recruiting moiety.^23^ *In vitro*, it elicited dose- and time-dependent degradation of LRG1 in HUVECs, achieving a DC_50_ of 14 μM, and significantly impairing key angiogenic processes, including endothelial cell motility, migration, and tube formation. In mice with alkali-burned corneas, ^**ET**^ **TAC-2** demonstrated marked efficacy in suppressing CNV progression, reducing both LRG1 expression within neovascularized corneal tissues and the release of pro-angiogenic factors through inhibition of the TGF-β-Smad1/5/9 signaling pathway.

**Figure 8:**
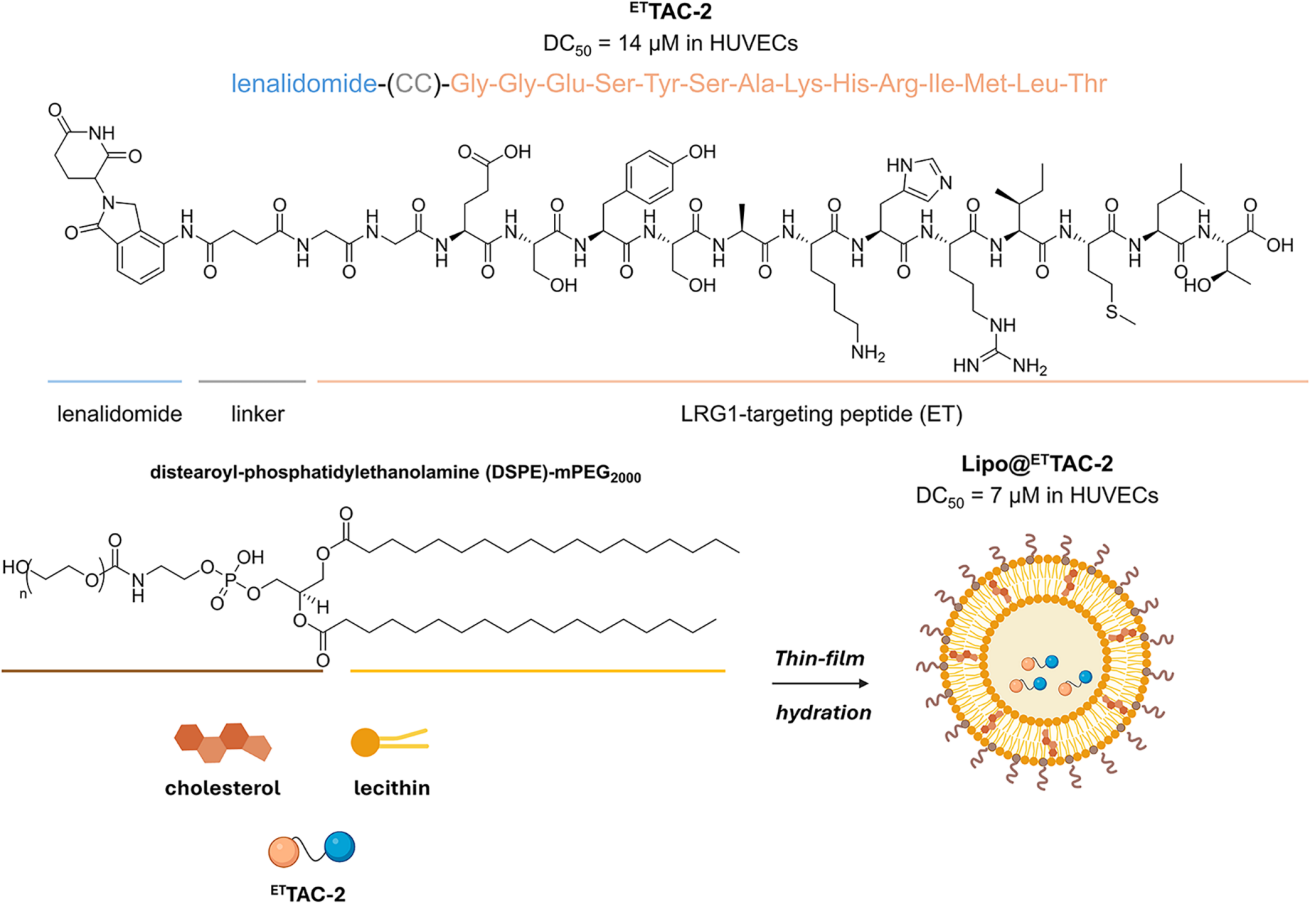
Design and formulation of ^**ET**^ **TAC-2** into liposomes (**Lipo@** ^**ET**^ **TAC-2**).

To enhance corneal bioavailability and prolong ocular retention, ^**ET**^ **TAC-2** was encapsulated into liposomes (**Lipo@** ^**ET**^ **TAC-2**), a clinically validated nanocarrier system, via thin-film hydration using optimized ratios of lecithin, cholesterol, and distearoyl-phosphatidylethanolamine (DSPE)-PEG_2000_ (Figure 8). The formulation achieved high encapsulation efficiency (76%) and drug loading (11%) at a 1:8 mass ratio of drug to lipids. Following topical administration, **Lipo@** ^**ET**^ **TAC-2** enhanced corneal adherence and facilitated efficient transcorneal penetration via both endocytosis and paracellular pathways. Accumulating within the corneal stroma and neovascular endothelial cells, **Lipo@** ^**ET**^ **TAC-2** released ^**ET**^ **TAC-2** intracellularly, thereby initiating the ubiquitination and subsequent degradation of LRG1. Notably, **Lipo@** ^**ET**^ **TAC-2** demonstrated a dose-dependent capability for targeted degradation of LRG1, significantly outperforming the degradation capacity of its unencapsulated form with a DC_50_ of 7 μM in HUVECs. It also demonstrated prolonged precorneal retention time and superior therapeutic outcomes in CNV models under a four-times-daily administration regimen.^22^

## Conclusions

Localized delivery of PROTACs to skin, ocular, or respiratory tissues offers a distinct translational advantage: it can achieve potent local target knockdown while markedly reducing systemic exposure, thereby uncoupling on-target efficacy from systemic liabilities that might limit systemically dosed degraders. This principle is already supported by emerging clinical data for topical AR degraders: **GT20029** met its phase II primary end point for AGA and acne, according to results reported by Kintor Pharma in 2024. The PROTAC, applied as a gel or tincture, showed a favorable safety/tolerability profile with no treatment-related sexual adverse events, providing early clinical proof-of-concept that local degradation can deliver efficacy while preserving systemic safety. Additional preclinical and early clinical examples illustrate how local degradation can translate into clinically meaningful improvements versus canonical topical inhibitors. In preclinical models, emerging topical JAK-targeting PROTACs have shown stronger local anti-inflammatory effects than topical ruxolitinib, with evidence of reduced systemic immune activation in preclinical readouts, an observation that supports the concept that local degradation of signaling kinases can outperform occupancy-based inhibition. Local degradation is also relevant in cancer therapy, where systemic administration of cytotoxic agents is often limited by severe adverse effects. While orally administered vepdegestrant has demonstrated that systemic PROTAC therapy can accomplish effective and generally well-tolerated target degradation in breast cancer, localized delivery strategies are also emerging. In this context, microneedle-mediated administration of **ERD-308**, which produced potent local ER degradation without systemic toxicity in preclinical models, might offer an alternative approach to enhance tolerability and patient compliance in suitable indications.

However, despite these unequivocal advantages, the translational promise must be balanced by realistic hurdles.

### Systemic Exposure

Topical administration does not equate to being risk-free, and unexpected systemic exposure and side effects may still occur. For example, the recently approved topical ruxolitinib carries warnings similar to those associated with oral JAK inhibitors.^53^ Additionally, reported cases of sexual dysfunction following the use of topical finasteride highlight the importance of comprehensive pharmacokinetic and toxicological evaluations.^54^ Hence, robust dermal, ocular, and pulmonary PK studies are required to confirm the therapeutic window observed in preclinical studies, as exemplified by phase I clinical trials of **AH-001**, which highlight the need for careful PK and tolerability assessments across different concentrations (0.2%, 0.5%, 1%, and 2%).

### Target Site Reaching and Retention

There are currently no universal physicochemical criteria defining the “ideal” properties of a topical PROTAC, since optimal features, such as MW, clogP, TPSA, solubility, and p*K*_a_, depend strongly on the route of administration and the intended site of action (e.g., epidermis, dermis, or subcutaneous tissue), as well as on the specific target, tissue barriers, and therapeutic context. Therefore, rather than pursuing a single optimal profile that fits all cases, the rational development of topical PROTACs relies on an integrated strategy, where each candidate is designed on a case-by-case basis by combining molecular design, property evaluation, formulation optimization, and tailored delivery platforms.

### Targeted Delivery

Topical administration minimizes the exposure of distant tissues but does not guarantee the targeting of only disease-relevant cell types or microenvironment. Hence, high local concentrations near heterogeneous cell populations necessitate careful target-selectivity profiling and histological assessment to exclude unintended degradation in bystander cells. Microneedle platforms and nanoparticle carriers might achieve focused deposition (e.g., **Se-AR PROTAC**-loaded HA-based microneedles in hair follicle niches). They concentrate drug payloads in the relevant microanatomical compartments, improving the therapeutic index compared with simple topical tinctures or creams. However, these delivery platforms present trade-offs (manufacturing/regulatory complexity, potential local irritation, or inflammation) that must be addressed in development plans. Complementary directions in terms of molecular design to further circumscribe the activity of topical PROTACs are currently being explored: (i) soft PROTAC design to further preserve local activity, while reducing systemic half-life; (ii) optically controlled PROTACs,^55^ which may enable spatiotemporal control of degradation; and (iii) development of predictive in silico tools for retention of bifunctional degraders in the site of action.

### Dose Selection

Due to the hook effect, local high concentrations may paradoxically reduce efficacy. Translational programs must, therefore, incorporate local PD measurements via tissue biomarkers to identify the optimal therapeutic dose range. Moreover, integrating spatial distribution models specific to dermal, ocular, or pulmonary compartments will further refine the dose selection and translation from preclinical to clinical settings.

### Immunogenicity and Metabolism

Peptide-based constructs or nanoparticle conjugates may carry additional risks, including local immune responses or proteolytic degradation that alters the exposure and activity.

### Microbiome Interactions

Potential interactions with local microbiota, particularly in cutaneous and ocular applications, should be assessed, as they may affect degrader stability or local immune responses.

### Formulation Stability And Compatibility

Physicochemical optimization of PROTACs for formulation stability, particularly in emulsions, gels, and nanoparticles, remains a critical determinant of translational success.

### Regulatory Complexity

Depending on the regulatory context (e.g., EU vs US), microneedles and other delivery systems may be regulated as medicinal products, medical devices, or combination products, requiring early consultation with regulatory agencies.

In summary, topical PROTACs represent a translationally distinct therapeutic modality whose clinical value depends on the combined optimization of chemistry, tailored formulations, and early translational studies that assess local pharmacodynamics with minimal systemic exposure. While topical delivery does not eliminate all risks, it shifts the risk–benefit balance in favor of local indications, where chronic treatment or narrow systemic windows would otherwise limit systemic use. Future clinical programs should integrate comprehensive PK/PD studies, toxicology, reproducible niche-targeting delivery systems, and early engagement with regulatory authorities to maximize the translational success of this promising therapeutic class.

## Abbreviations Used

AD: atopic dermatitis
AGA: androgenetic alopecia
AR: androgen receptor
BET: bromodomain and extra-terminal domain
BRD4: bromodomain-containing protein 4
CDK4/6: cyclin-dependent kinase 4 and 6
CNV: corneal neovascularization
CRBN: cereblon
DC_50_: half-maximal degradation concentration
DHU: dihydrouracil
DNFB: 2,4-dinitrofluorobenzene
ERα: estrogen receptor α
HA: hyaluronic acid
IL: interleukin
IPF: idiopathic pulmonary fibrosis
JAK: Janus kinase
LRG1: leucine-rich α-2-glycoprotein 1
MITF: microphthalmia-associated transcription factor
MRT: mean retention time
MW: molecular weight
O/W: oil-in-water
PROTAC: proteolysis-targeting chimera
QSPR: quantitative structure–property relationship
STAT: signal transducer and activator of transcription
TPSA: topological polar surface area
TYK2: tyrosine kinase 2
TYR: tyrosinase
UPS: ubiquitin–proteasome system
VHL: von Hippel–Lindau
